# Portable Raspberry Pi Platform for Automated Interpretation of Lateral Flow Strip Tests

**DOI:** 10.3390/s26020598

**Published:** 2026-01-15

**Authors:** Natalia Nakou, Panagiotis K. Tsikas, Despina P. Kalogianni

**Affiliations:** Department of Chemistry, University of Patras, GR26504 Patras, Greece

**Keywords:** lateral flow assay, strip, beads, polystyrene microparticles, SARS-CoV-2, Python, image analysis, image processing, OpenCV, DNA

## Abstract

Paper-based rapid tests are widely used in point-of-care diagnostics due to their simplicity and low cost. However, their application in quantitative analysis remains limited. In this work, a nucleic acid lateral flow assay (NALFA) was integrated with an automated image acquisition system built on a Raspberry Pi platform for the quantitative detection of SARS-CoV-2 virus, increasing the accuracy of the test compared to subjective visual interpretation. The assay employed blue polystyrene microspheres as reporters, while automated image capturing, image processing and quantification were performed using custom Python software (version 3.12). Signal quantification was achieved by comparing the grayscale intensity of the test line with that of a simultaneously captured negative control strip, allowing correction for illumination and background variability. Calibration curves were used for the training of the algorithm. The system was applied for the analysis of a series of samples with varying DNA concentrations, yielding recoveries between 84 and 108%. The proposed approach integrates a simple biosensor with an accessible computational platform to achieve full low-cost automation. This work introduces the first Raspberry Pi-driven image processing approach for accurate quantification of NALFAs and establishes a foundation for future low-cost, portable diagnostic systems targeting diverse nucleic acids, proteins, and biomarkers.

## 1. Introduction

Point-of-care testing (POCT) encompasses the development and application of advanced analytical devices and platforms designed to facilitate rapid diagnostics, characterized by operational simplicity, portability, and either disposability or reusability. Among them, paper-based sensors are one of the most significant platforms for POCT. Lateral flow assays (LFAs) and paper-based microfluidic devices have demonstrated significant utility in diagnostics. In particular, self-tests demonstrated their critical role during the COVID-19 pandemic. LFAs consist of a diagnostic membrane on which proper biorecognition molecules are immobilized at a test line and where accumulation of color nanoparticles occurs in the presence of the analyte, forming a visual color line. The most significant limitation of LFAs is that they provide only semi-quantitative results. Therefore, advancing LFAs toward quantitative analysis with high adaptability and flexibility is of great importance. Integrated systems must be developed to ensure proper usability even by unexperienced users [1]. Another limitation of LFAs is the potential of subjective interpretation of the visual test results, which can lead to diagnostic inaccuracies. This is particularly crucial when the test line appears extremely faint, increasing the likelihood of false negative assessment [2]. Automated analysis of the visual outcomes of the results based on advanced image analysis tools is thus required to provide qualitative, as well as quantitative, results with enhanced accuracy, eliminating the problems of false interpretation based on personal bias. Simplicity is also important to allow applicability for point-of-care testing (POCT) and enable testing in the field or in remote areas. In this context, Raspberry Pi has been recently exploited as an important tool for analytical appliances, providing automated analysis of various experimental data. Open-source hardware has attracted the interest of researchers in different scientific fields, giving the opportunity to develop simple and cost-effective laboratory instruments and devices and making such instrumentation affordable and accessible for many researchers and users. Raspberry Pi, as a low-cost single-board computer, has become very popular for a variety of applications. Raspberry Pi is inexpensive and easy to program, compared to smartphones. Few attempts have also been made for the integration of Raspberry Pi with LFAs and microfluidics devices. Raspberry Pi has been used for controlling pumps and the flow and temperature of flowing systems. The integration of Raspberry Pi with microfluidics has transformed the landscape for the development of portable, low-cost, highly customized and flexible devices. However, in biomolecular diagnostics, image analysis is also a key parameter. For this reason, improved Raspberry Pi platforms have been integrated with Raspberry Pi camera modules to allow for automated image capturing and subsequent image processing [3,4].

In more detail, a smartphone and a Raspberry Pi portable reader were used for the detection of progesterone in milk in paper-based microfluidic devices. Two LEDs were used for excitation, while Raspberry Pi was used to control the LEDs. A smartphone application based on OpenCV was developed for image processing. The proposed reader showed better performance when compared to the traditional ELISA assay [5]. Raspberry Pi was also exploited for the automatic calculation of Michaelis–Menten enzyme kinetics on different paper substrates on portable devices [4], to monitor the flow rate in paper-based microfluidic devices to determine blood coagulation [6,7] and in a flexible fluoropolymer microcapillary film for blood testing related to blood response to stimulation [8]. A microfluidic immunobiosensor was also developed for the detection of *Salmonella*. The detection was based on a metal–organic framework that had peroxidase activity and catalyzed the colorimetric oxidation of o-phenylenediamine, which was monitored by Raspberry Pi [9]. A paper-based microfluidic device with a hand-held Raspberry Pi detection system was also presented for the determination of the albumin-to-creatine ratio in human urine via colorimetric reactions in two parallel channels. The method was in good agreement with a benchtop method [10]. Another paper-based microfluidic chip was developed for capillary flow velocity profile determination for screening of oil types using Raspberry Pi and machine learning [11]. A novel microfluidic chip was developed in which the entire diagnostic assay was performed, including sample loading, nucleic acid extraction, and reverse transcription–loop-mediated isothermal amplification (RT-LAMP) reaction and detection. The device had four reaction chambers and was applied for the detection of four respiratory viruses. Fluorescent measurements of the RT-LAMP reactions were performed with a Raspberry-Pi-based detection system using a Sony IMX2198 megapixel CMOS sensor (Tokyo, Japan) and a five-parameter log-logistic (5PLL) curve for signal intensity determination [12]. A 3D-printed instrument was also constructed using a Raspberry Pi camera and an easy-to-use LabVIEW software-based (version 17.0.1fl) program for automated image analysis and real-time monitoring of colorimetric LAMP reactions [13]. More recently, a portable microfluidic in vitro diagnostic device was developed for the rapid detection of procalcitonin based on silver nanoparticles, and a 3D-printed detection device was constructed including a microscope lens and a Raspberry Pi camera for image acquisition. Images were then manually processed based on RGB analysis using ImageJ software [14].

Regarding LFAs, a fluorescent lateral flow immunoassay strip together with a fluorescence reader based on Raspberry Pi was developed for the detection and quantitation of C-reactive protein for inflammation monitoring using fluorescence-labeled detection antibodies. The optical system comprised two lasers that were controlled by the Raspberry Pi, while the emitted fluorescence was captured by a Raspberry Pi camera Module v2 NoIR that had a Sony IMX219 sensor. A black strip holder was constructed, and image analysis was conducted using Python. The computer could also perform the image analysis and communicate the results to a mobile phone application acting as a user interface [15]. An automated algorithm using a Raspberry Pi based on Python and OpenCV through the python package ’RPi.GPIO 0.7.0’ was also developed for the quantitation of commercial colorimetric paper-based immunosensors, including LFAs, for the detection of SARS-CoV-2 antibodies. A special 3D-printed box was constructed to hold the paper sensors, and an LED was used for light measurements. The proposed method, compared to manual measurements, minimized the risk of false positive or false negative results [16]. Another Raspberry Pi-based reader was constructed for the quantitative analysis of immune-LFAs with gold nanoparticles as reporters for the detection of digoxin in human serum. PP-LiteSeg, an image segmentation neural network, was developed as a part of the PaddlePaddle Neural Network Library for image processing. After analyzing seven samples with various concentrations of digoxin, the root-mean-squared error was 20.7% [17]. Finally, a nanozyme-based lateral flow immunoassay was developed for the detection of H1N1 viral protein combined with automatic centrifugal microfluidics. The detection system included a Raspberry Pi, its camera and a Qt-based software for algorithm development. The system provided a lower LOD compared to that obtained by the naked eye and semi-quantitative analysis [18]. There are few other approaches for the quantitative analysis of LFAs that are mainly based on the use of smartphones and integrated with specific smartphone applications in some cases. CMOS sensors or digital cameras have also been used. Most of these applications, however, have not performed detailed quantitative analysis other than calibration curve construction [19,20,21,22,23,24,25]. In more detail, lateral flow immunoassays have been developed in combination with a smartphone and a smartphone application for the detection of allergens in milk using a commercially available LFA [26], human serum albumin, alkaline phosphatase in milk, saliva cortisol and TSH using a smartphone application in Matlab 2018b and R2010a [27,28,29,30] and IgG and IgM antibodies for SARS-CoV-2 using Python 3 and the PyQt5 library [25]. Dark boxes or 3D-printed accessories were also used to enable image acquisition [19,31,32]. OpenCV and machine learning were also exploited for quantification purposes [19]. A CMOS camera was also utilized for image acquisition, and post-processing image analysis was performed using the OpenMV H7 camera module that can run MicroPython [24]. For automated quantification within NALFAs, smartphones and smartphone applications were also used for the analysis of HIV [33], while a smartphone-based system consisting of a 3D-printed photo box for standardized positioning and lighting, a smartphone for image acquisition and a R Shiny open-source package were also developed for the automated analysis of LFAs [23].

We herein report the development of a nucleic acid-based lateral flow assay (NALFA) employing colored polystyrene nanoparticles as reporters for the molecular detection of SARS-CoV-2 virus. The NALFA was then integrated with an automated image analysis platform comprising a Raspberry Pi, a Raspberry Pi camera and a Python-based algorithm, enabling both qualitative and quantitative analyses of the test strips. To the best of our knowledge, this is the first Raspberry Pi-driven automated image processing system for the analysis of optical outputs from NALFAs utilizing colored polystyrene microparticles for signal generation. Colored polystyrene microparticles exhibit better stability in buffers and more rapid conjugation than gold nanoparticles, the most used reporter in LFAs, while they have also shown advanced discrimination capability in multiplex LFAs integrated with automated image analysis using smartphones [34,35,36].

## 2. Materials and Methods

### 2.1. Materials, Software and Instrumentation

The reagents used in this study include the following: 1-Ethyl-3-(3-dimethylaminopropyl) carbodiimide (EDC) (AppliChem, Maryland Heights, MO, USA), 2-(N-Morpholino)ethanesulfonic acid (MES), methanol, sucrose, Tween-20, Tris(hydroxymethyl)aminomethane (Tris-Base) and sodium dodecyl sulfate (SDS) from Sigma–Aldrich (Saint Louis, MO, USA), biotinylated polyadenine oligonucleotide (b-dA(30)) (VBC Biotech, Vienna, Austria), oligonucleotides (Eurofins Ebersberg, Germany), deoxynucleotide triphosphates (dNTPs) (Invitrogen, Carlsbad, CA, USA), Triton-X-100 (Thermo Fisher Scientific, Waltham, MA USA), Kapa2G Fast PCR Kit (Kapa Biosystems, Basel, Switzerland), ethylenediaminetetraacetic acid (EDTA) and NaCl from Merck (Darmstadt, Germany), glycerol (Carlo Erba, Barcelona, Spain), disodium phosphate (Na_2_HPO_4_) (Lachner, Neratovice, Czech Republic), streptavidin (SA) (Roche, Basel, Switzerland), terminal deoxynucleotidyl transferase (TdT) (New England Biolabs, Ipswich, MA, USA), carboxylated blue polystyrene microspheres with a diameter of 0.197 μm (Bangs Laboratories Inc., Fishers, TN, USA), and a nitrocellulose membrane, an immersion pad, a conjugate pad and absorbent material from Cytiva (Marlborough, MA, USA). The primers, the detection DNA probe and the amplified DNA sequence of SARS-CoV-2 are presented in Table 1.

The instrumentation and devices include a Raspberry Pi 4 Model B, 4 GB RAM, 2018 and the Raspberry Pi Camera Module 2 (8 MP, lens 3280 × 2464 px) (Raspberry Pi Trading Ltd., Cambridge, UK) for image capturing and analysis of the strips’ images. The Dice TP60 thermal cycler (Takara Bio, Kusatsu, Japan) was used for SARS-CoV-2 DNA amplification and hybridization reactions. The automative dispenser Linomat 5 (Camag, Muttenz, Switzerland) and the UVP CrossLinker CL-3000 (Analytik Jena, Jena, Germany) were used for strip construction. The software employed in this study included Image J version 1.54h for densitometric analysis, OpenCV for image processing and Picamera2 library for image acquisition.

Finally, 1× PBS Buffer, pH 7.4, constituting 137 mM NaCl, 2.7 mM KCl, 8 mM Na_2_HPO_4_ and 2 mM KH_2_PO_4_, and 6× SSC buffer, pH 7.0, constituting 0.9 M NaCl and 0.09 M sodium citrate, were used. The composition of 1× TE buffer was 10 mM Tris-HCl pH 8.0 and 1 mM EDTA, that of the running buffer was 1× PBS pH 7.4, 0.1% (*w*/*v*) Triton-X-100 and 0.05% (*w*/*v*) SDS and that of the hybridization buffer was 220 mM NaCl, 110 mM Tris-HCl, pH 8.0 and 0.088% Triton-X-100. The diluents for both poly(dA), used in the construction of the control zone, and streptavidin, used in the construction of the test zone, consisted of 5% (*v*/*v*) methanol, 2% (*w*/*v*) sucrose, and 6× SSC buffer, pH 7.0.

### 2.2. Coupling of Beads with Poly(dT) Oligonucleotide

For the modification of carboxylated microspheres, 10 μL of microspheres were added to 100 μL of MES buffer (0.1 M, pH 4.5). The microspheres were sonicated for 5 min and then centrifuged at 7000× *g* for 10 min. The microspheres were washed twice with 100 μL of MES buffer and resuspended with 200 μL of the same buffer. Resuspension was enabled by vortexing and sonication for 5 min. Then, 10 μL of a freshly prepared EDC solution (10 mg/mL) was added, and the activation of the carboxylated microspheres was carried out for 15 min at room temperature in the dark. Subsequently, 1 μL of amino-modified thymidine oligonucleotide (NH_2_-dT(30)), at a concentration of 100 pmol/μL, and another 10 μL of EDC solution (10 mg/mL) were added, and the mixture was incubated for 1 h under the same conditions. Next, 2 μL of 10% (*v*/*v*) Tween-20 were added to the modified microspheres, and the suspension was centrifuged for 10 min at 7000× *g*. This was followed by two additional washing steps with 200 μL of 1× TE buffer, pH 8.0, and 1 μL of 10% (*v*/*v*) Tween-20. A final resuspension was performed in 85 μL of 1× TE buffer, pH 8.0, for storage or further use.

### 2.3. Addition of Poly(dA) Tail to Oligonucleotides

A tailing reaction was performed to add a poly(dA) tail to the DNA probe specific to the PCR product of SARS-CoV-2 or a random oligonucleotide used for the construction of the control zone of the rapid test. The reaction mixture contained 1× TdT buffer, 0,25 mM CoCl_2_, 400 pmol of the DNA probe or 1000 pmol of the random probe, 2 mM or 5 mM of dATP, respectively, and 30 U of terminal deoxynucleotidyl transferase (TdT). The mixture was incubated for 1 h in a water bath at 37 °C. Finally, 2 μL of 500 mM EDTA (pH 8.0) were added to terminate the reaction, followed by storage at −20 °C.

### 2.4. Amplification of the Target Sequence via Polymerase Chain Reaction (PCR)

The SARS-CoV-2 DNA sequence was amplified by PCR in a final volume of 20 μL. The PCR mixture contained 1× Kapa 2G Fast Ready Mix, 1 pmol of the forward primer, 1 pmol of the biotinylated reverse primer, and 1 μL of the SARS-CoV plasmid of a concentration of 10^8^ DNA copies/μL. The PCR parameters were as follows: initial denaturation at 95 °C for 3 min, and then 35 cycles of denaturation at 95 °C for 5 s, primer annealing at 60 °C for 20 s and primer extension at 72 °C for 5 s, followed by a final extension at 72 °C for 1 min.

### 2.5. Hybridization of PCR Products to Complementary Probe

The PCR products were hybridized to 1 pmol of a complementary DNA probe containing a poly(dA) tail, as described in Section 2.3. Hybridization was performed in a thermal cycler at 95 °C for 2 min and at 37 °C for 10 min.

### 2.6. Construction of the Rapid Strip Test

The lateral flow assay (LFA)–rapid strip, with dimensions of 4 mm × 70 mm, consisted of four parts: the immersion pad, the conjugate pad, the nitrocellulose diagnostic membrane, and the absorbent pad. Beneath all these components, an adhesive plastic backing was placed, which provided stable support for the individual segments. The overlapping assembly of these components was essential to ensure the continuous flow of the running buffer along the strip through the development of capillary actions. For the construction of the test line and the control line on the diagnostic membrane of the strip, a poly(dA) sequence (2.5 pmol/strip) was deposited onto the control zone and streptavidin (SA) (2.4 μg/strip) was deposited onto the test zone using the Linomat 5 (CAMAG) semi-automatic dispenser at a speed of 100 nL/s. The membrane was then placed into the UVP CrossLinker CL-3000 and dried for 5 min at an energy of 125 mJ/cm^2^. The lateral flow test strip was then assembled as follows: the membrane was placed first on the adhesive plastic supporting material, followed by the conjugate pad and the absorbent pad, positioned below and above the membrane, respectively. These two components must slightly overlap the membrane to support continuous flow of the reagents. Finally, the immersion pad was placed at the bottom of the strip, slightly overlapping the conjugate pad. Once all components were assembled, the assembly was cut into 4 mm wide strips.

### 2.7. Nucleic Acid-Based Lateral Flow Assay

Volumes of 5 μL of the hybridized PCR product (with the complementary DNA probe), 5 μL of microspheres conjugated with poly(dT) sequences, and 10 μL of the running buffer were applied to the conjugate pad of the strip. The strip was then immersed in 320 μL of the running buffer (1× PBS buffer (pH 7.4), 0.1% (*w*/*v*) Triton-X 100, and 0.05% (*w*/*v*) SDS). The strip was removed from the solution after 10–15 min, and the PCR products were visually detected. Finally, images of the biosensors were captured using the Raspberry Pi Camera Module 2 and stored on the Raspberry Pi 4 for further analysis.

### 2.8. Image Acquisition and Image Processing Based on Raspberry Pi—Development of a Home-Made Image Processing Algorithm Based on Python

Image Acquisition: the Raspberry Pi Camera Module 2 was connected via the CSI interface and positioned at a fixed optimum distance from the strips. The strips were illuminated with uniform ambient lighting to minimize shadows and reflections. The image acquisition process was automated using the Picamera2 Python library by development of a home-made and Python-based algorithm. The camera was initialized with a full-resolution still configuration, and after a brief warm-up delay, a high-resolution JPEG image was captured and stored locally on the Raspberry Pi. Image Processing: the captured image was processed using the OpenCV library and converted to grayscale to simplify analysis. Three specific regions of interest (ROIs) were defined using fixed pixel coordinates as follows: (i) Control zone: the upper line on the membrane of the strip test was used to confirm the test’s validity. (ii) Test zone: the lower line on the membrane was used to evaluate the presence of the analyte, while the intensity of the color of the line indicated the analyte’s concentration. (iii) Reference zone: a negative strip test was placed next to the strip test and analyzed simultaneously in the same picture, so that the white background area of the negative strip at the test zone was used as a neutral brightness reference. Then, the average grayscale value of each ROI was calculated using NumPy version 2.2.0. The grayscale intensity of the control zone was measured first to ensure the validity of the test. A test was considered valid only if the control zone had an average grayscale value above a predefined threshold (e.g., 30). Tests without a valid control zone were excluded from the analysis. For the valid tests, the grayscale difference Δy was calculated between the test and the reference zone as below:

Δy = ∣Gtest − Greference∣

where G denotes the average grayscale intensity of each zone.

Finally, the software displayed the following on a PC screen for each test: (a) the average grayscale value of the control zone (for validity check), (b) the average grayscale values of the test and reference zones, (c) the difference Δy between the test and reference zones, (d) the estimated concentration x of the analyte in fmol, (e) the mathematical model used based on the calibration of the system, and (f) the validity status of the test. Additionally, the software saved the original captured image and the cropped grayscale images of the test, the control, and the reference zone on the Raspberry Pi.

## 3. Results

**Figure 1:**
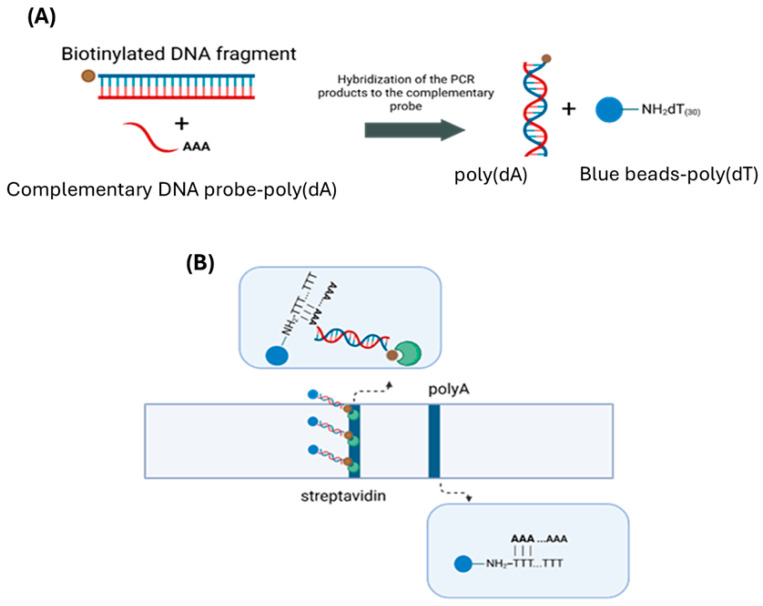
(**A**) Hybridization reaction of the PCR product for SARS-CoV-2 to the complementary DNA probe. (**B**) Principle of the lateral flow assay.

In this study, a molecular rapid test was developed and integrated with a Raspberry Pi and a Raspberry Pi camera for the automated detection and quantification of the SARS-CoV-2 virus. Colored polystyrene microspheres were used as reporters for signal generation. An automated ‘reading’ of the visual colored lines of the strips was developed, enabling analysis and minimizing the human error due to subjective interpretation of the results. For method development, a plasmid containing the DNA sequence responsible for the expression of the nucleoprotein of SARS-CoV-2 was used as a mοdel accounting for real sample analysis. This sequence was amplified by PCR, quantified via agarose gel electrophoresis, and subsequently, after hybridization, detected by the molecular rapid test. The PCR products were biotinylated and had a length of 72 base pairs. Hybridization was performed using a specific DNA probe that carried a poly(dA) tail at its 3′ end (Figure 1A). The hybrids were deposited onto the conjugate pad of the strip together with the poly(dT)–conjugated polystyrene microspheres. The strip was then immersed in the running buffer, and as the liquid migrated upwards, all reagents drifted along the strip. A blue test line formed as the biotinylated hybridized products bound to the streptavidin and immobilized onto the membrane at the test zone of the strip, due to the strong and specific biotin–streptavidin interaction. The poly(dA) tail of the hybrids was hybridized to the poly(dT) sequence of the blue-conjugated microspheres, leading to accumulation of the microspheres at the test zone, producing a visible signal. A second blue line, corresponding to the control line of the strip, was formed as the excess of the blue poly(dT)-conjugated microspheres were captured by immobilized poly(dA) sequences at this area of the strip (Figure 1B).

Finally, images were captured using the Raspberry Pi Camera Module 2, processed using GIMP 2.10.38, and analyzed via a custom Python code. An integrated automated system was therefore developed for the automated reading and quantification of the test line of the strips, thereby determining the concentration of the PCR product. The objective of this project was to develop a low-cost, user-friendly and automated image processing system for the detection and determination of the concentration of a target biomolecule (e.g., SARS-CoV-2 virus) using a rapid test integrated with a Raspberry Pi-based imaging system. The grayscale intensity difference between two defined regions (a test and a reference region) in a captured image of the strips was used to estimate the concentration of the target through calibrated mathematical models.

### 3.1. Optimization Studies

**Figure 2:**
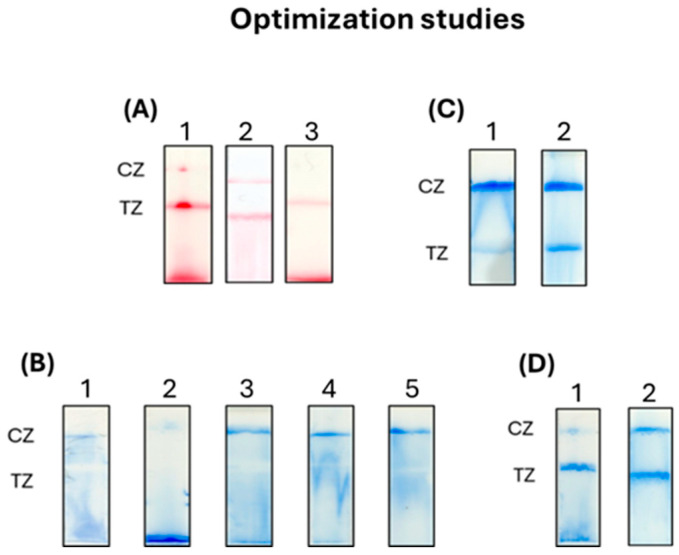
(**A**) Optimization of the hybridization assay with thermal denaturation and hybridization in 1× PCR buffer (1), in hybridization buffer (2) and with NaOH-assisted denaturation (3). (**B**) Optimization of the composition of the running buffer: (1) 1× PBS pH 7.4, 0.5% Tween-20, 0.05% SDS, (2) 1× PBS pH 7.4, 0.5% Tween-20, 0,05% SDS, 0.5% BSA, (3) 1× PBS pH 7.4, 0.05% SDS, 0.2%Triton-X-100, (4) 0.1% Triton X-100, (5) 1.0% Triton X-100. (**C**) Different approaches of the application of the sample and the beads onto the conjugation pad of the strip: (1) pre-mixing of the PCR product with the beads and (2) pre-mixing of the PCR product with the running buffer and the beads prior to the application to the strip. (**D**) Different compositions of the running buffer for positive strips: (1) 1× PBS pH 7.4, 0.05% SDS, 0.2% Triton-X-100 and (2) 1× PBS pH 7.4, 0.05% SDS, 0.1% Triton-X-100. Red zones correspond to beads of red color and blue zones are formed using blue-colored beads. CZ: control zone, ΤZ: test zone.

The development of the proposed lateral flow strip using colored polystyrene microparticles was based on previous studies [34,35,36]. Further optimization studies, however, were performed in order to obtain the most intense color signals at the test line of the strip, in combination with high specificity and good flow of the microspheres along the strip, for the molecular detection of SARS-CoV-2. Optimization studies included the hybridization reaction, the composition of the running buffer and the application of the sample to the strip test for analysis. Initially, two hybridization reactions were tested: (i) thermal denaturation and (ii) NaOH-assisted denaturation of the double-stranded amplified products with subsequent hybridization to the DNA probe. For the first reaction, a volume of 2 μL of the PCR product was mixed with 1 μL of the DNA probe—poly(dA) (1 pmol/μL), 1 μL of 900 mM NaCl and 6 μL of 1× PCR buffer or hybridization buffer (220 mM NaCl110 M Tris-HCl pH 8.0, 0.088% Triton-X-100). The mixture was incubated for 2 min at 95 °C and for 10 min at 37 °C. For the NaOH-based denaturation, a volume of 2 μL of the PCR product was mixed with 1 μL of the DNA probe—poly(dA) (1 pmol/μL), 3 μL of 1× PCR Buffer and 5 μL of NaOH 0.4 Μ,and the mixture was left for denaturation for 5 min at room temperature. Then, 5 μL of HCl 0.4 Μ and 5 μL of Tris-HCl 0.4 Μ pH 7.5 were added, followed by incubation for 15 min at room temperature to allow hybridization. The hybrids were applied to the strip for analysis, along with 5 μL of red beads–poly(dT) conjugates. The images of the strips are presented in Figure 2A. Thermal denaturation provided more efficient hybridization, as it provided the most intense color at both the test and the control line of the strip. A volume of 5 μL of the PCR product was also tested for hybridization following the thermal denaturation protocol, but with no better results.

The running buffer was then optimized. The running buffer is crucial as it affects the flow of the beads along the strip, the clearance of the membrane, the efficiency of the hybridization of complementary DNA sequences and the elimination of non-specific bindings. For these reasons, different compositions of the running buffer were tested in order to obtain the best flow of the beads and the optimum clearance of the diagnostic membrane, forming an intense blue zone at the control zone with the lowest non-specific bindings at the test zone of the strip. The results are presented in Figure 2B. PBS buffer provides a suitable ionic strength for efficient hybridization between two DNA strands and interaction of biotin moieties with streptavidin, while Tween-20 and Triton X-100 are surfactants that allow good flow of the beads and minimize non-specific bindings and SDS eliminates non-specific interactions. With the use of the running buffer 1 (1× PBS pH 7.4, 0.5% Tween-20, 0.05% SDS), we observed that the beads were not captured at the control zone, while the use of 0.5% BSA (Bovine Serum Albumin) in running buffer 2 resulted in aggregation of the beads, which could not migrate along the strip. In the three following running buffers (3–6), we replaced Tween-20 with Triton-X-100 in various concentrations (0.1, 0.2 and 1.0%). Triton-X-100 provided better flow of the beads. However, during the analysis of positive samples containing amplified PCR products, the flow of the beads was again hindered. For this reason, we tested different application approaches of the sample and the beads onto the conjugation pad. In the first approach, the hybridized PCR product was mixed with the beads and incubated for 10 min at room temperature before its application to the strip. For the second approach, the hybridized PCR product was mixed with 10 μL of running buffer and then with the beads, and the mixture was applied to the strip. The second approach gave better flow along the strip (Figure 2C). We again tested the use of Triton-X-100 in the running buffer at 0.1 and 0.2% with that of 0.1%, giving better results (Figure 2D).

### 3.2. Detectability of the Rapid Test

After optimization, we constructed a calibration curve in order to assess the detectability of the system. Initially, we tested the efficiency of the conjugated beads by constructing a calibration curve using a biotinylated single-stranded target, a biotinylated poly(dA) (b-dA(30)) sequence, using different concentrations of the target ranging from 0 to 250 fmol. As low as 1.56 fmol of the single-stranded DNA target was detectable by the rapid test (Figure S1).

### 3.3. Development of the Automated Image Processing System Based on Raspberry Pi and Python—Algorithm Description

**Figure 3:**
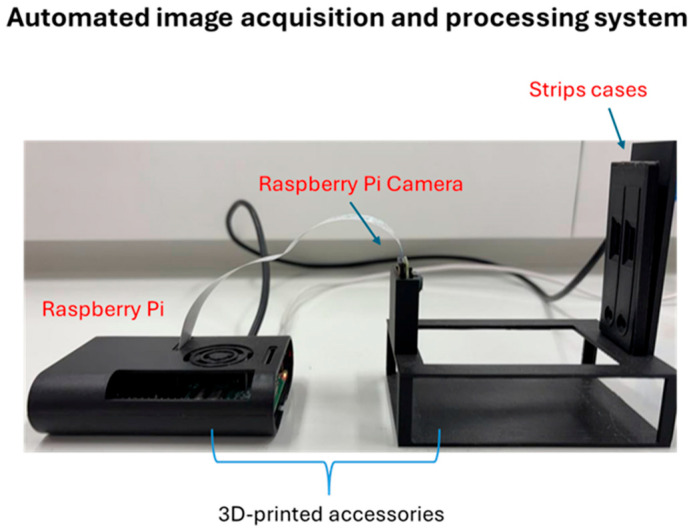
The automated image acquisition and processing system that comprises the Raspberry Pi, the Raspberry Pi camera, the 3D-printed strip cases for the test and the reference strip, and the 3D-printed accessories used to mount the whole platform.

Initially, a 3D-printed image acquisition system was designed and fabricated, as shown in Figure 3, fixing the following together: the Raspberry Pi, special strip cases for the strips to be analyzed and the Raspberry Pi camera at a fixed position from the strips in order to acquire an image of high quality. The strip cases were designed accordingly to fit two strips (a reference and a test strip) exactly in terms of width and length and hold it firmly in the same position, as well as to contain windows where both the test and control lines of each strip are visualized in a fixed position. In the context of developing an automated system for detecting and quantifying the intensity of the optical signal on rapid strip tests using a Raspberry Pi and a home-made algorithm developed in Python, a series of successive tests were conducted to enable reliable discrimination between positive and negative samples, as well as to estimate the concentration of the amplified product. However, for more accurate quantification, simultaneous analysis of a test sample and a negative sample within the same image was selected to establish a fixed reference point for the corresponding background. The negative strip test corresponds to the negative sample of the amplification reaction to ensure more accurate quantification. For this purpose, two 3D-printed black cases were designed, constructed and positioned together in the 3D-printed image acquisition system (Figure 3). A quantification algorithm was then designed in Python to (a) distinguish between negative and positive samples and (b) in the case of positive samples, correlate the optical signal with the amount of analyte. To determine the difference in the grayscale intensity between the test zone of the test sample and the corresponding area of the negative sample (where no color signal appears), a relative intensity value was calculated and used for relative quantification. For high accuracy, all photographs were taken with the camera mounted in a fixed, calibrated position, maintaining constant distance, angle, and lighting conditions. In this context, a stable camera stand and a white background were used to ensure a consistent brightness level and uniform contrast across all images. Simultaneously, the diagnostic strips were placed with a specific and reproducible orientation in the suitable strip cases (Figure 3). This ensured that the predefined cropping of the images coordinated in the software was performed accurately to isolate the control and test zones of the strips. Any deviation in strip positioning or scaling could alter the geometry of the regions of interest (at the pixel level), leading to incorrect grayscale intensity measurements and unreliable quantification results. As mentioned above, to minimize the impact of external factors, test samples were photographed simultaneously with negative strip tests within the same image. This concurrent capture ensured that both samples were subjected to identical lighting conditions and camera settings, enabling reliable comparison of the zones and relative quantification of the signal based on grayscale intensity differences. Even if image acquisition conditions varied slightly between different samples, comparison within the same image remained reliable, since the measurement was based on relative signals to corresponding signals of negative controls.

**Figure 4:**
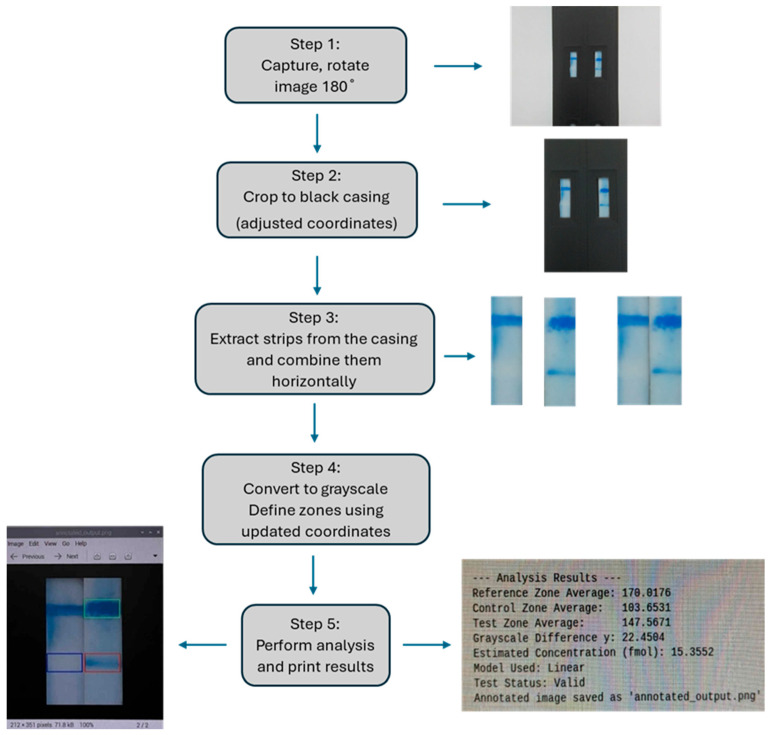
The workflow of the image processing Python-based algorithm.

The developed quantification algorithm was implemented in Python; it performed a series of steps, starting from image acquisition and concluding with the estimation of the analyte’s concentration. In summary, the algorithm workflow includes the following stages (Figure 4): (1) Image acquisition using the Raspberry Pi camera (Picamera 2) under standardized distance, angle and lighting conditions. (2) Image rotation and cropping to isolate the region containing the two strip tests. (3) Extraction of specific regions (reference, test, and control zone) based on predefined coordinates. (4) Conversion to grayscale and measurement of the mean intensity value for each region. (5) Calculation of intensity difference (Δy) between the test and the reference zone. (6) Application of a mathematical model to determine the target quantity (x), expressed in fmol. The model selection depends on the signal range. (7) Validation of result reliability based on the numerical solution. (8) Visualization and storage of the final image, including graphical frames marking the analyzed regions and displaying the output values—intensity measurements and estimated concentration. Image analysis and numerical computations were implemented in Python using the OpenCV library (for image processing) and NumPy (for numerical operations and data array manipulation). The accuracy and consistency of the grayscale analysis and the mathematical modeling performed by the developed software largely depend on the standardized image acquisition procedure. More details about algorithm development are described in the Supplementary Material.

### 3.4. Qualitative and Quantitative Analysis of the Rapid Tests Through Grayscale Signal Analysis

#### 3.4.1. Calibration of the System

**Figure 5:**
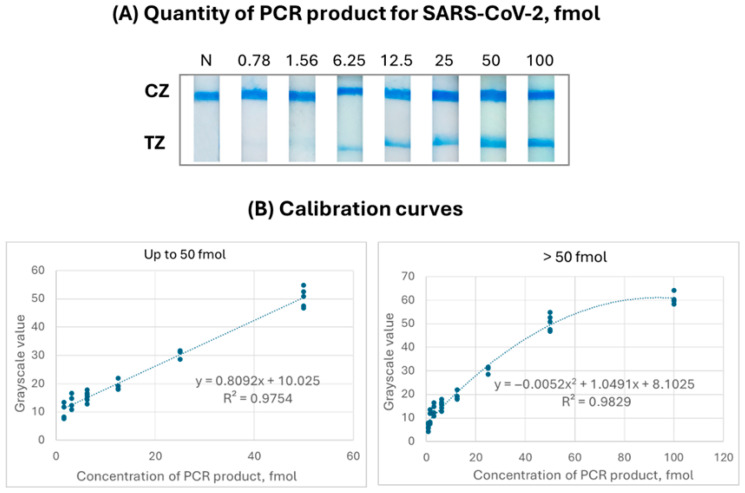
(**A**) Calibration curves of different concentrations of the PCR product analyzed with the rapid test. (**B**) The calibration graphs of the grayscale values of the test zone of each strip versus the amount of PCR product for quantification with the developed system. CZ: control zone, ΤZ: test zone.

First of all, calibration of the developed algorithm was performed in order to obtain accurate quantitative results. For this purpose, calibration curves were constructed by preparing successive dilutions of the PCR product for SARS-CoV-2 in 1× PCR buffer (10 mM Tris-HCl, pH 8.4, 500 mM KCl) ranging from 0 to 100 fmol, which were analyzed with the rapid test (Figure 5A). The construction of the calibration curves of the target was repeated 6 times to increase the quantitative accuracy of the test (Figure S2). As low as 0.78 fmol of the PCR product was detectable by the molecular strip test. For each strip test, the difference in grayscale intensity of the colored test line between the test and a corresponding negative test was calculated. These grayscale values were used as the optical response on the *y*-axis of the calibration curve, while the corresponding analyte concentrations were plotted on the *x*-axis. For the calibration of the quantification system, two models were employed: a linear model for PCR product concentrations up to 50 fmol, and a quadratic model for higher concentrations, where signal saturation occurs (Figure 5B). The first calibration curve was used to estimate the concentration of unknown samples based on a grayscale intensity difference (Δy) of ≤47.559 relative to the negative reference sample within the same image, while the second calibration curve was applied to higher target concentrations, where signal saturation occurs and the linear correlation is no longer adequate.

#### 3.4.2. Application of the Automated System for the Analysis of a Series of Samples

**Figure 6:**
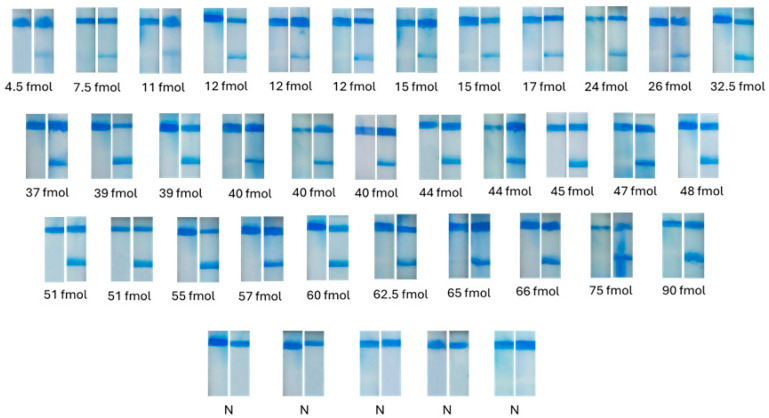
Analysis of a series of samples with the rapid test and the developed algorithm. N: negative.

To evaluate the accuracy and reliability of the automated image processing system for detection and quantification of rapid tests, randomly selected PCR product samples with various concentrations, as well as negative samples, were analyzed with the rapid test (Figure 6). For each sample, the following parameters were recorded: the grayscale intensity value of the test line, the estimated concentration (as determined by the model), the actual concentration of the PCR product, the % relative error, and the % recovery. In cases where samples did not contain the analyte, the grayscale difference between the reference strip and the negative strip is very small or close to zero. In such instances, the concentration calculated from the calibration equation may yield a negative or zero value, and the sample is classified as negative. Although negative concentrations have no physical meaning, they are interpreted as non-detectable analyte and are categorized as negative samples. Following the analysis of a series of negative samples, a threshold grayscale difference of <8.1025 units was established. Samples with Δy values below this threshold were considered negative and were not subjected to further quantification by the algorithm. After quantitative analysis of the samples, the calculated % relative error and the % recovery were 2–16% and 84–108%, respectively, for the linear equation and 3–17% and 85–107%, respectively, for the quadratic equation. Both mathematical models exhibited very good accuracy. Moreover, as observed from the calibration curves and from the analysis of various samples, the system had a resolution of 1–2 fmol of analyzed PCR product related to SARS-CoV-2. Saturation of the signal at the test line of the strip occurs above 50 fmol of PCR product.

### 3.5. Repeatability of the Automated Integrated System

The repeatability assessment was performed by calculating the coefficient of variation (%CV) for each concentration, based on the grayscale values obtained from the algorithm for six independent runs. The results showed that at low concentrations (0.78–3.125 fmol), the %CVs ranged between 17.9% and 27.7%, indicating relatively higher variability, which is acceptable at concentrations close to the limit of detection. In contrast, at medium and high concentrations (≥6.25 fmol), the %CV values ranged from 4.2 to 8.6%, proving the very good repeatability of the whole system. The variability at low concentrations was estimated by analysis of a series of samples with different techniques that revealed a logarithmic relationship between the variability of chemical measurements and the concentration of the analyte which was independent of the analyte and of the technique used for measurement. At low concentrations, the signal is very small and random fluctuations (noise) from the instrument or environment have a significant influence on the signal, leading to variable results and thus lower precision. Moreover, for this application, the use of a professional dispenser, rather than a research-based one, for the construction of the sensing areas of the strip may lead to a more reproducible method of strip construction, increasing the overall precision, even at low concentrations [37,38,39].

## 4. Discussion and Conclusions

An automated system for the detection and quantitative estimation of nucleic acids—specifically targeting the SARS-CoV-2 virus—was developed through the integration of nucleic acid-based lateral flow assay (NALFA) technology with automated image analysis implemented in Python on a Raspberry Pi platform. Despite the widespread use of advanced image processing tools, their application in the quantitative interpretation of lateral flow assays/rapid tests remains limited. The developed system enabled the detection of colored sensing zones on rapid tests employing blue microspheres as reporters. Quantification was achieved by measuring the grayscale intensity of the test line of each strip, thereby allowing for the relative quantification of the analyte concentration in comparison with a negative control (reference sample). Following optimization studies, calibration curves were constructed for system calibration. The inclusion of an internal reference—specifically, a negative control strip whose image was captured simultaneously with the test strip—facilitated correction for environmental and optical variations such as lighting and background intensity, thus enhancing measurement accuracy. A dedicated software tool was developed in Python, utilizing the OpenCV and NumPy libraries, to detect and compute intensity differentials between test and reference regions and apply either a linear or quadratic mathematical model for concentration estimation based on the provided calibration. In this approach, the sensing zones of the strip are localized by the algorithm by first detecting the control line. The fixed ROIs were designed to be slightly larger than the visible regions of the band. This ensures that, even if minor positional shifts occur, the entire test line remains within the ROI, thereby preventing signal loss or misalignment and securing simplicity, low computational cost, and speed for the Raspberry Pi platform. In addition, the image is pre-cropped around the strips prior to ROI computation, which significantly reduces the likelihood of erroneous ROI measurements. This step constrains the sensing zones of the strip to a smaller, controlled frame, thereby avoiding errors arising from large images containing noise or irrelevant regions. Therefore, small differences in lateral flow strips from different baches, e.g., different lengths of the strips, do not affect localization. Moreover, the cassette into which each strip is inserted is properly designed to hold the strip in a reproducible, constrained position to ensure the accurate detection of the zones. Finally, the presence of random materials in the area may affect the result; however, due to the size of the region, the impact will be very small. This minimizes the need for dynamic detection and avoids potential errors from automatic alignment algorithms (e.g., due to noise, reflections, or minor strip imperfections). Experimental evaluation using 40 samples with varying target DNA concentrations demonstrated high accuracy, reliability and repeatability, with recoveries ranging from 84 to 108% and relative errors below 20%. Moreover, the system achieved 100% sensitivity, specificity, and accuracy in qualitative classification of positive and negative samples. Other approaches based mainly on smartphones and developed applications, usually using Python, CMOS sensors and digital cameras, provided recoveries ranging between 84 and 110% [19,20,21,22,23,24,25,26,27,28,29,30,31,32,33]. We therefore have introduced a compact integrated platform of lower cost and have reduced subjective errors using the corresponding equipment, compared to smartphone-based approaches for automated image acquisition and image processing, for both qualitative and quantitative analysis of NALFAs, providing similar accuracy compared to the existing methods. A detailed quantification of samples with various analyte concentrations was also performed.

The key innovation of this approach lies in the integration of a low-cost biosensing platform with inexpensive and accessible computational technology, enabling automated and accurate quantification of rapid tests without the requirement for specialized laboratory equipment or trained personnel. The incorporation of a fixed reference point and grayscale-based comparative analysis ensured measurement robustness under variable illumination conditions. The proposed integrated system provides a portable, user-friendly and field-deployable diagnostic tool that overcomes limitations due to subjective interpretation of the visual outcomes of LFAs. Continuous refinement of the algorithm through expanded calibration datasets is expected to further enhance quantitative accuracy. The described system establishes a foundation for the development of analogous diagnostic platforms for diverse analytes, including infectious agents, biomarkers, or other nucleic acid sequences. Furthermore, the diagnostic platform could also be extended to lateral flow immunoassays for the automative detection/quantification of antigens and proteins, promoting low-cost diagnostic solutions overcoming the requirement of complex and costly laboratory-based systems.

## Figures and Tables

**Table 1:** Sequences of synthetic oligonucleotides.

Oligonucleotide	Sequence (5′ → 3′)
Forward Primer (SARS-CoV-2)	GACCCCAAAATCAGCGAAAT
Reverse Primer (SARS-CoV-2)	Biotin-TCTGGTTACTGCCAGTTGAATCTG
SARS-CoV-2 Target Sequence	GACCCCAAAATCAGCGAAATGCACCCCGCATTACGTTTGGTGGACCCTCAGATTCAACTGGCAGTAACCAGA
SARS-CoV-2 Detection Probe	ACCCCGCATTACGTTTGGTGGACC

## Data Availability

Dataset available on request from the authors.

## Supplementary Materials

The following supporting information can be downloaded at: https://www.mdpi.com/article/10.3390/s26020598/s1, Figure S1: Calibration curve of single-stranded DNA sequence (b-dA(30)) with the nucleic acid-based rapid test. N: negative, CZ: control zone, TZ: test zone; Figure S2: Calibration curves of different concentrations of the PCR product for SARS-CoV-2 with the nucleic acid-based rapid test. N: negative, CZ: control zone, TZ: test zone.

## Abbreviations

The following abbreviations are used in this manuscript:

LAMP	Loop-mediated isothermal amplification
LFAs	Lateral flow assays
MES	2-(N-Morpholino)ethanesulfonic acid
NALFA	Nucleic acid lateral flow assay
PCR	Polymerase chain reaction
POCT	Point-of-care testing
ROI	Region of interest
RT	Reverse transcription
SDS	Sodium dodecyl sulfate
TdT	Terminal deoxynucleotidyl transferase
