# Supplementary material for: Portable Raspberry Pi Platform for Automated Interpretation of Lateral Flow Strip Tests
Source: Sensors (Basel). 2026 Jan 15;26(2):598. doi: 10.3390/s26020598 (PMC12845611; doi:10.3390/s26020598)
Supplement: Supplementary file 1 [file sensors-26-00598-s001.zip › sensors-4071035-supplementary.pdf]

Supplementary material

Portable Raspberry Pi Platform for Automated Interpretation of Lateral Flow Strip Tests

Natalia Nakou, Panagiotis K. Tsikas* and Despina P. Kalogianni*

Department of Chemistry, University of Patras, GR26504 Rio, Patras, Greece

*Corresponding authors
Despina P. Kalogianni
E-mail: kalogian@upatras.gr
Panagiotis K. Tsikas
panostsikas@upatras.gr

Development of the automated image processing system based on Raspberry Pi and Python – Algorithm and image processing description

The calibration and quantification procedure includes the following phases:

1. ***Calibration Phase.*** Initially, a series of samples with known concentrations (from 0 to 100 fmol) were analyzed for system calibration. For each known concentration, the grayscale intensity difference Δy between the Test Zone and the corresponding Test Zone (white area) of a negative control strip, that was simultaneously analyzed and photographed, was calculated. The calibration process was repeated to ensure greater accuracy.

2. ***Model Development.*** The Δy was correlated with the known concentration of the analyte (x, in fmol) and calibration graphs were generated. For low-to-medium concentrations ($\Delta y < 47.559$), the relationship was linear. For higher concentrations, the relationship was better described by a quadratic equation due to saturation of the test line of the lateral flow strip.

3. ***Algorithm Implementation.*** Two mathematical relationships were created, and these equations were integrated into the algorithm.

4. ***Analysis of Unknown Samples.*** Finally, for each random/unknown sample, the algorithm automatically calculates the Δy values from the images of the two strips (the test and the negative control), selects the appropriate model (linear or quadratic) based on the Δy value, applies the corresponding equation, and calculates the estimated concentration of the analyte in fmol.

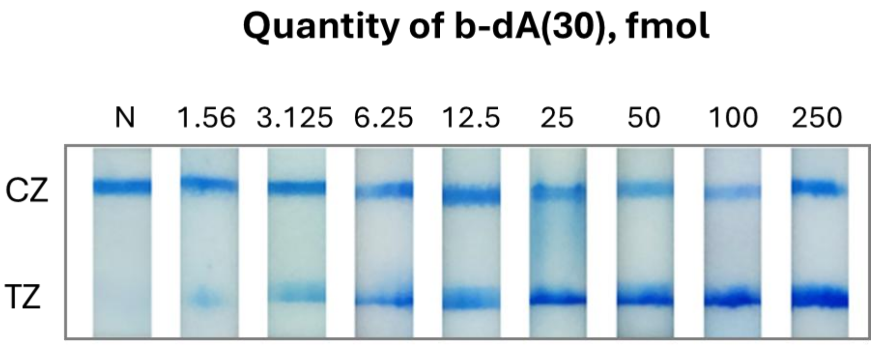

Figure S1. Calibration curve of single-stranded DNA sequence (b-dA(30)) with the nucleic acid-based rapid test. N: negative, CZ: control zone, TZ: test zone.

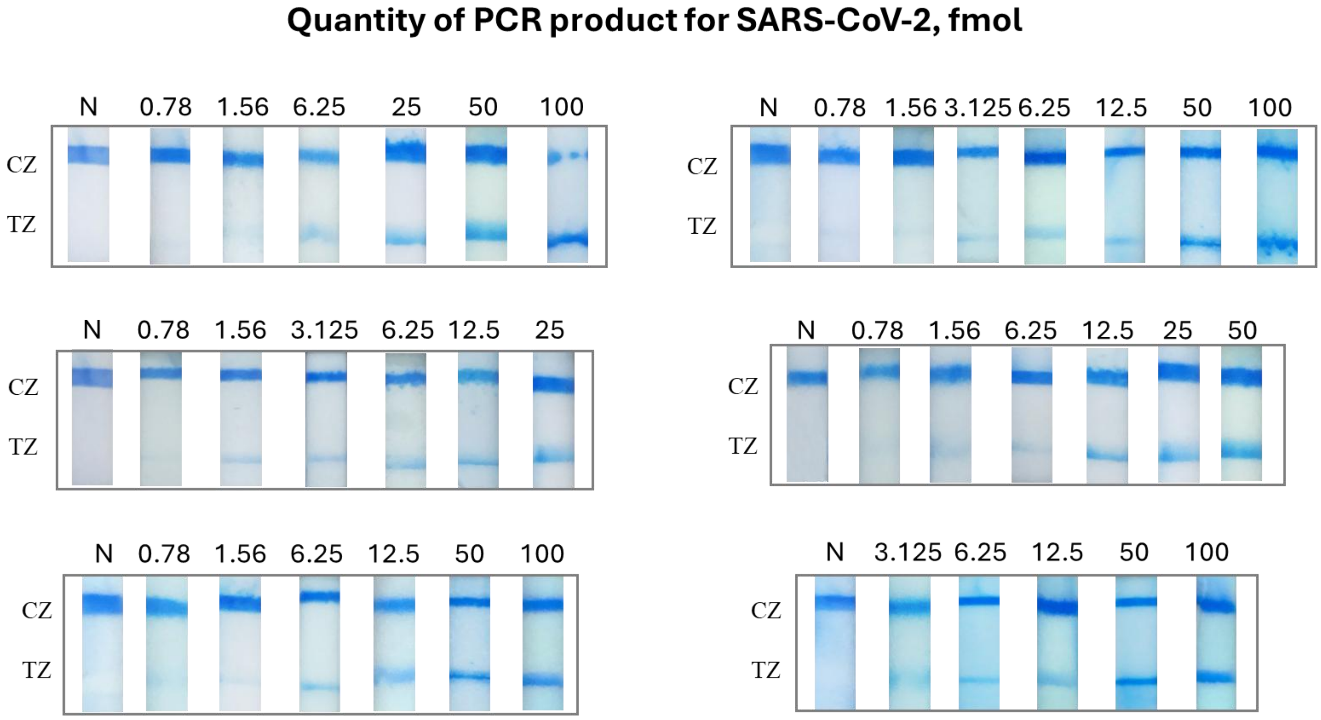

Figure S2. Calibration curves of different concentrations of the PCR product for SARS-CoV-2 with the nucleic acid-based rapid test. N: negative, CZ: control zone, TZ: test zone.
